# Reproductive natural history and successful juvenile propagation of the threatened Caribbean Pillar Coral *Dendrogyra cylindrus*

**DOI:** 10.1186/s12898-015-0039-7

**Published:** 2015-03-16

**Authors:** Kristen L Marhaver, Mark JA Vermeij, Mónica M Medina

**Affiliations:** CARMABI Foundation, Piscaderabaai z/n, PO Box 2090, Willemstad, Curaçao; School of Natural Sciences, University of California at Merced, 5200 North Lake Road, Merced, CA 95343 USA; Aquatic Microbiology, Institute for Biodiversity and Ecosystem Dynamics, University of Amsterdam, Science Park 700, 1098 XH Amsterdam, The Netherlands; Current Address: Department of Biology, Pennsylvania State University, 324 Mueller Lab, University Park, PA 16802 USA

**Keywords:** Coral reproduction, Early life history, Spermcasting, Larvae, Threatened species, Coral reefs, Pillar coral, *Dendrogyra cylindrus*, Caribbean

## Abstract

**Background:**

The Caribbean pillar coral *Dendrogyra cylindrus* was recently listed as a threatened species under the United States Endangered Species Act. One of the major threats to this species is its low, virtually undetectable recruitment rate. To our knowledge, sexually-produced recruits have never been found in over 30 years of surveys of Caribbean reefs. Until recently, the reproductive behavior of *D. cylindrus* was uncharacterized, limiting efforts to study its early life history, identify population bottlenecks, and conduct outplanting projects with sexually-produced offspring. In Curaçao, we observed the spawning behavior of this species over three years and five lunar cycles. We collected gametes from spawning individuals on three occasions and attempted to rear larvae and primary polyp settlers.

**Results:**

Here we describe successful fertilization methods for *D. cylindrus* and we document rapid embryonic development. We describe the successful propagation of embryos to the swimming larvae stage, the first settlement of larvae in the laboratory, and the survival of primary polyp settlers for over seven months. We show that spawning times are highly predictable from year to year relative to the lunar cycle and local sunset times. We use colony-level data to confirm that males begin spawning before females. We also provide the first reports of split-spawning across months in this species.

**Conclusions:**

Together, our findings of consistent spawning times, split-spawning, rapid embryonic development, and remarkable robustness of larvae and settlers now enable expanded research on the early life history and settlement ecology of *D. cylindrus*. This will help biologists to identify the population bottlenecks in nature that underlie low recruitment rates. Further, the settlement of *D. cylindrus* larvae in the laboratory now makes out-planting for restoration more feasible. Asynchronous spawning times and rapid embryonic development may have important consequences for population biology, connectivity, and management, by affecting fertilization dynamics and larval dispersal distances. We argue that a precautionary approach to conservation is warranted, given this species’ peculiar life history traits and still-unresolved population structure. Overall, the natural history and husbandry contributions presented here should facilitate accelerated research and conservation of this threatened coral.

**Electronic supplementary material:**

The online version of this article (doi:10.1186/s12898-015-0039-7) contains supplementary material, which is available to authorized users.

## Background

**Figure 1:**
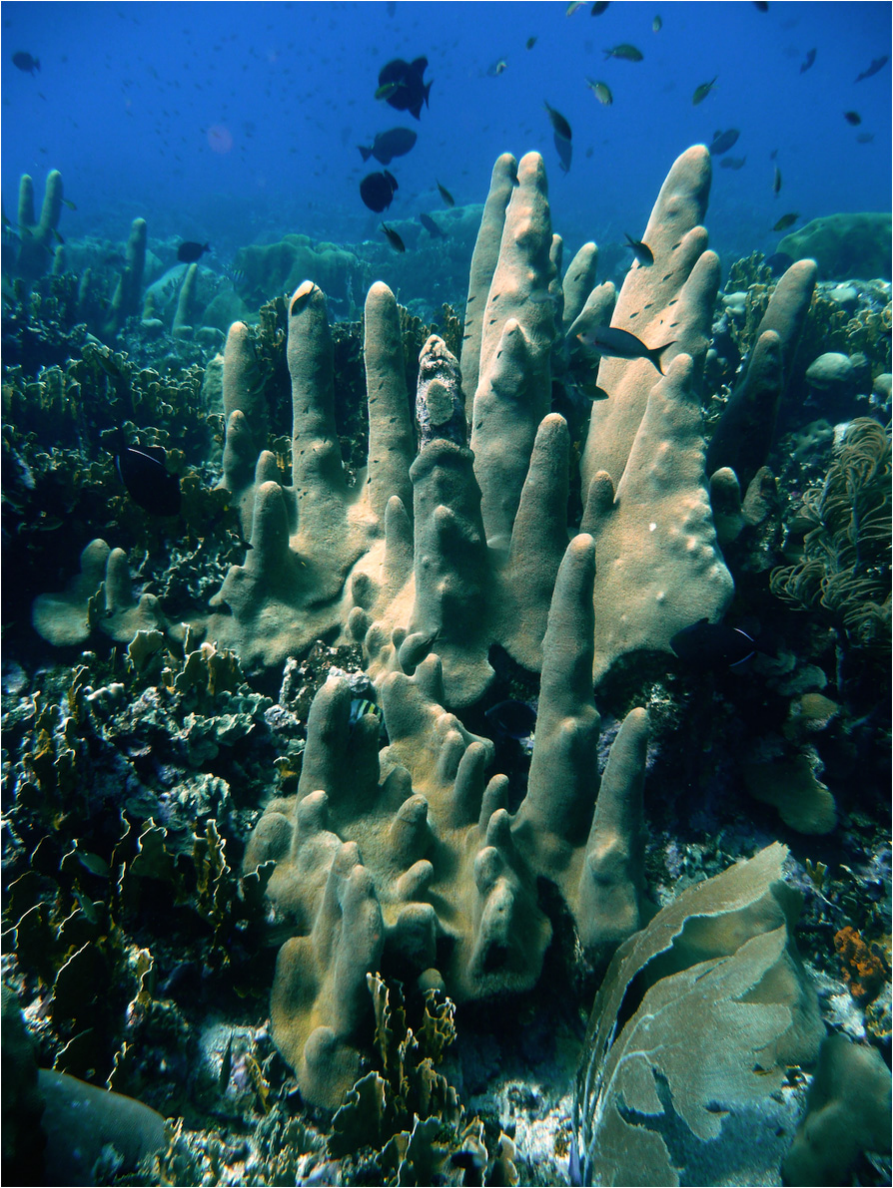
**The threatened Caribbean Pillar Coral** ***Dendrogyra cylindrus*** **.** The pillar morphology of *D. cylindrus* is unique among Caribbean coral species. Colonies can reproduce asexually by fragmentation of pillars, which re-attach to the reef and grow new vertical pillars (foreground).

The pillar coral *Dendrogyra cylindrus* Ehrenberg 1834 (Figure 1) was recently listed as a threatened species under the United States Endangered Species Act [1]. As an uncommon species whose range is limited to the Caribbean, this coral has been understudied by scientists [2]. The resulting lack of knowledge has hindered conservation [3], inhibited research on reproduction and early life history, and limited options for restoration projects. As the only Caribbean coral that forms tall vertical pillars, and as the only species in its genus, *D. cylindrus* warrants enhanced conservation concern because it is morphologically and evolutionarily unique (e.g., [4-6]). Limiting studies of its early life history, the reproductive timing of *D. cylindrus* was unknown for many decades [7] and then known only through histological inference [8] until 2006, when a single male was observed spawning [9]. Mass spawning of multiple males and females was first documented in August 2012 [10].

Despite progress in identifying the timing of reproduction, information on the developmental biology, larval biology, and juvenile recruitment ecology of *D. cylindrus* has remained elusive. These are particularly critical life history stages because, for unknown reasons, zero *D. cylindrus* settlers or recruits have been found in large-scale surveys across the Caribbean, including in Curaçao (1975 and 2005; [11,12]), the U.S. Virgin Islands (1980s; [13]), the Florida Keys (1999–2009; [14]), coastal Colombia (2002; [15]), and Puerto Rico (2003–2005; [16]). The absence of sexually-produced juveniles does not appear to be readily explained by simply a lack of adult colonies. For example, in Curaçao, groups of approximately 20 to 200 *D. cylindrus* colonies occur in dense stands at multiple points along the leeward coast of the island. These stands typically occur at depths of 3–8 m, on prominent, rocky outcrops with high wave exposure. Individual colonies also regularly occur on straight sections of the leeward coast with solid limestone basements and a consistent directional current, typically at 5–8 m depth (K. Marhaver, unpublished data). Though isolated stands of adult *D. cylindrus* persist, there appears to be a severe population bottleneck during reproduction or dispersal. The cause and timing of this bottleneck remain unidentified.

Prior to our study, the consistency of spawning times across years was not known, characteristics of gravid colonies had not been described, methods for propagation were not developed, settlement surfaces were untested, and no primary polyp settlers had been observed or photographed on the reef or in the laboratory. Such a large knowledge gap slowed the study and possible conservation and restoration of this unique species. Our goal was therefore to fill as many knowledge gaps as possible by applying methods from our coral spawning research to the study of *D. cylindrus*.

## Results

### Timing of spawning

**Figure 2:**
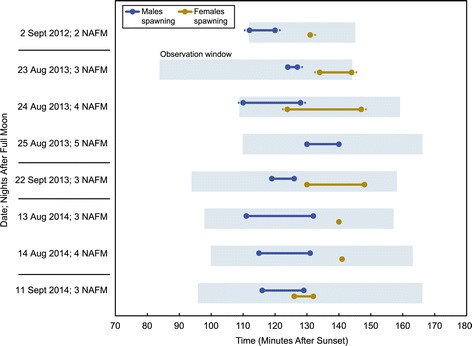
**Spawning times of male and female** ***D. cylindrus*** **colonies in Curaçao relative to local sunset time.** Blue rectangles depict the window of observation. Blue lines denote the time period when males were seen spawning. Yellow lines denote observations of females spawning. Dotted lines indicate probable spawning that was not observed because a diver arrived at or departed from a colony while it was spawning heavily. All times are presented as minutes after sunset (MAS) relative to the Willemstad, Curaçao sunset time on the night of observation.

In Curaçao, over three separate years and over five separate lunar cycles in the months of August and September, we observed *D. cylindrus* colonies spawning on nights two to five after the full moon, from 110 to 147 minutes after sunset (Figure 2 and Additional file 1). Across all nights and years, individual male colonies were observed spawning between 110 and 140 minutes after sunset. Individual female colonies were observed spawning between 124 and 147 minutes after sunset. Overall, the timing of spawning for this species was highly consistent from 2012 to 2014 and between the months of August and September. Some individual colonies were observed spawning on consecutive nights or in consecutive years. Omitting all such repeat observations from the data set, distinct male colonies were first observed spawning at 112, 116, 118, 119, 119, 120, 120, 121, 121, and 126 minutes after sunset. Individual female colonies were first observed spawning at 126, 130, 131, 132, 133, 134, and 142 minutes after sunset. Males therefore began spawning significantly earlier than females (p = 0.0005; Mann–Whitney U-test, one-tailed). Across all nights, male spawning began between 4 and 29 minutes prior to the start of female spawning. Females were never observed spawning unless males in the area had already been observed spawning.

### Spawning behavior and appearance

**Figure 3:**
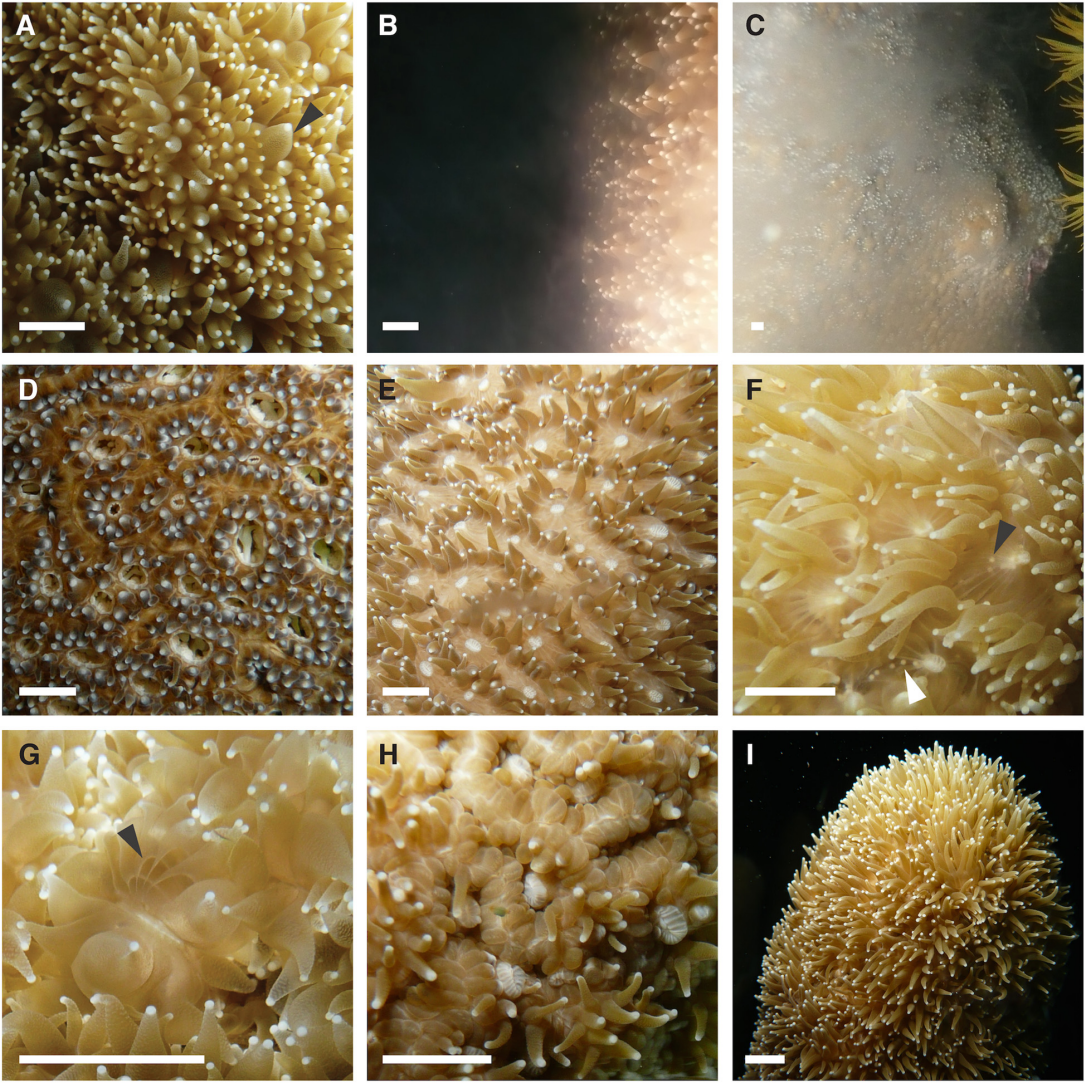
**Characteristics of** ***D. cylindrus*** **colonies before, during, and after spawning.** Male *D. cylindrus* **(A-C)** exhibited inflated tentacles prior to spawning (A; black arrowhead). Mouths were generally not visible. Male colonies released sperm directly into the water column in multiple pulses **(B-C)**. Tentacles were often retracted into the skeleton during and immediately after spawning **(C)**. After males began spawning, female *D. cylindrus* colonies **(D-I)** exhibited open mouths **(D)** and bloated tissues surrounding the mouth **(D-F)**. Eggs were visible prior to release (F; black arrowhead) and were in some cases exposed to ambient seawater through openings in the tissue (F; white arrowhead). Tissue openings were most apparent in a female with heavily-ballooned tentacles shortly after spawning **(G)**. After spawning, females often retracted tentacles into the skeleton **(H)**, however they typically returned to a normal appearance quickly thereafter **(I)**. All scale bars represent approximately 5 mm.

Before they released sperm, gravid males exhibited inflated tentacles that were extended away from their skeletons (Figure 3A). Individuals released sperm in multiple short pulses (Figure 3B-C). During and immediately after spawning, tentacles were often pulled tightly into the skeleton (Figure 3C). After spawning, tentacles no longer appeared inflated. After males began spawning, some female *D. cylindrus* colonies were observed with open mouths (Figure 3D) and others were observed with bloated tissues surrounding the mouth (Figure 3E-F). Eggs were visible inside these tissues, either arranged around the mouth or along open slits radiating away from the mouth (Figure 3F). In an extreme case, the ballooned tentacles of a female revealed that these openings in the tissue allow for extensive exposure to ambient seawater (Figure 3G). Females retracted their tentacles shortly after releasing eggs (Figure 3H). A few minutes later, female colonies returned to their typical appearance with tentacles extended and mouths no longer easily visible (Figure 3I).

### Fertilization, development, and larval survival

In August 2012, gametes were collected on the reef and mixed onshore. Embryos showed early signs of cell division but failed to complete development. In September 2013, gametes handled in the same manner did not show any signs of fertilization. In August 2013, fertilization was attempted on the reef. Approximately 30 eggs were collected from a tented female that was exposed to sperm in situ. These eggs were combined on shore with additional sperm and approximately 30 additional eggs that were collected in a separate region of the reef without the underwater mixing step. With this gamete pool, three different incubation methods were attempted using different fertilization times and seawater types: 1) fertilization for 20 minutes, transfer to fresh sperm solution for 90 minutes, then transfer to GF/F-filtered seawater, 2) fertilization for 20 minutes followed by transfer to filter-sterilized seawater (SSW), and 3) fertilization for 100 minutes followed by transfer to SSW. Overall, 45-48% of the eggs began cell division and successfully developed into larvae (7 of 15, 12 of 25, and 9 of 20 eggs in Treatments 1, 2, and 3, respectively). Seawater in the laboratory was held at ambient ocean temperature (~29°C).

**Figure 4:**
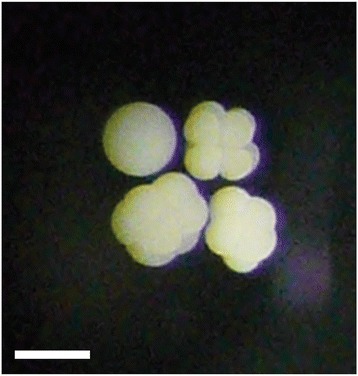
**Early development of** ***D. cylindrus.*** Shown are one unfertilized egg and three developing embryos at the eight-cell stage. Fertilized zygotes underwent holoblastic, equal cleavage through the 16-cell stage. The mode of gastrulation was not observed. Early cell divisions occurred at approximately 40 minute intervals. Embryos developed into swimming larvae in less than 16 hours. Scale bar represents approximately 0.5 mm.

Fertilized *D. cylindrus* embryos underwent holoblastic, equal cleavage through the 16-cell stage (Figure 4). Developing embryos were positively buoyant. Relative to the end of spawning, we first observed embryos at the 2-cell stage at 72 min, the 4-cell stage at 122 min, the 8-cell stage at 154 min, and the 16-cell stage at 182 min. These observations were made late in the 4-cell stage and early in the 16-cell stage; cell division therefore appears to occur at approximately regular 40-minute intervals during early development. We did not follow embryos overnight during subsequent divisions; we therefore did not observe gastrulation type or the timing of first movement.

On the day after spawning (Day 1), less than 16 hours after fertilization, embryos had developed into fully formed, swimming planula larvae. By this point, the majority of larvae were positively gravitactic, swimming in a directional manner at or near the bottom of the rearing containers, with occasional pauses. Larval survival was scored on Days 1, 4, 16, and 23 after spawning. The numbers of larvae alive in Treatments 1, 2, and 3, respectively, were as follows: Day 1) 7, 12, and 9; Day 4) 0, 4, and 4; Day 16) 0, 0, and 3. No larvae were found on Day 23.

### Settlement and post-settlement survival

**Figure 5:**
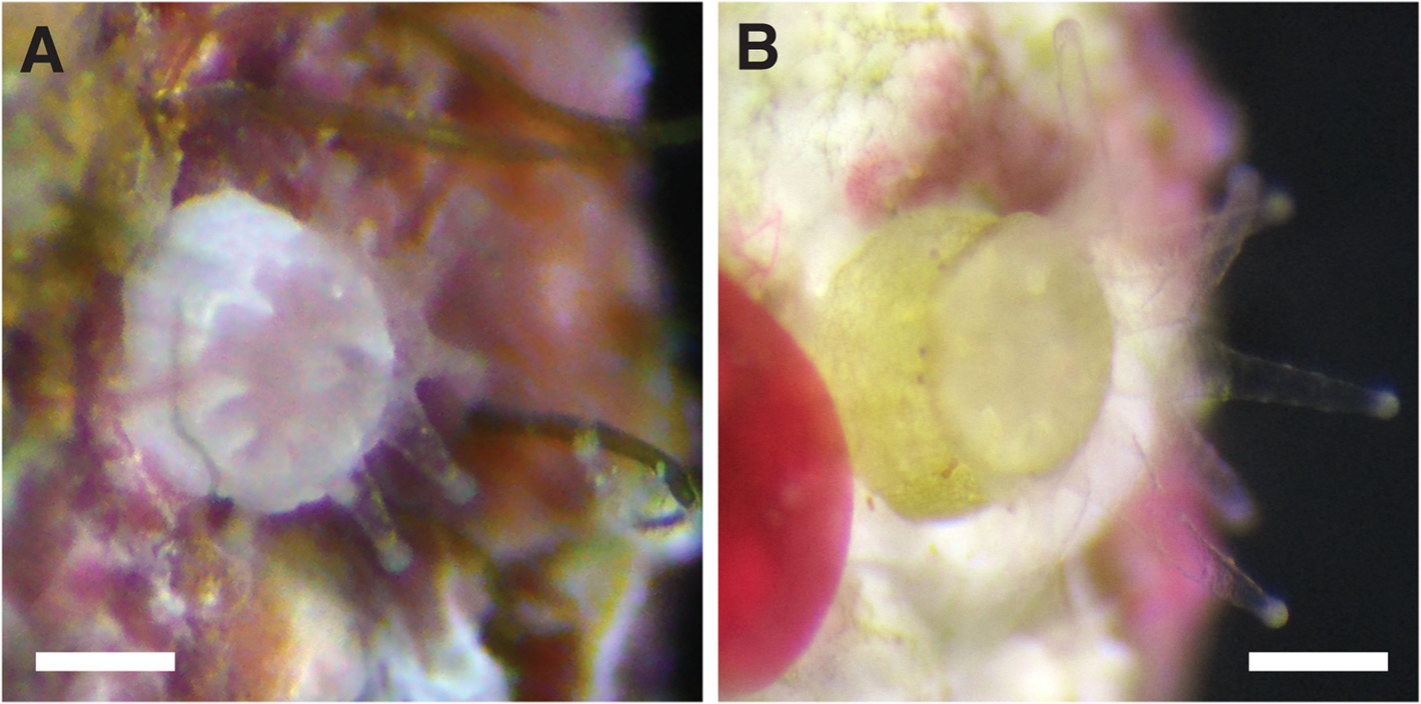
**Settled primary polyps of** ***D. cylindrus*** **.** A settled primary polyp 17 days after spawning **(A)** extends its characteristically large tentacles during the day, as do adults of this species. The same settler 77 days after spawning **(B)** shows growth of the tentacles and skeleton. This represents the first successful settlement and survival of *D. cylindrus* in the laboratory. Scale bars represent 0.5 mm.

On the fourth day following spawning, the first *D. cylindrus* settler was observed in Treatment 1 on the pre-cured surfaces (kiln stilts, i.e., ceramic tripods, which were pre-cured for two months in a flow-through aquarium system to develop communities of crustose coralline algae; Figure 5). In Treatment 2, a settler was found on the pre-cured surfaces on Day 16. In Treatment 3, a settler was found on the plastic (polystyrene) rearing container on Day 23. No settlement occurred on the un-cured ceramic surfaces. Despite the small numbers of settlers, this first achievement of *D. cylindrus* settlement in the laboratory nevertheless represents 8-14% of the starting number of larvae from the three incubation treatments.

Notably, primary polyp settlers exhibited characteristics typical of adult *D. cylindrus*: large tentacles in proportion to corallite size and tentacles extended during the day. The skeletal cup also featured a prominently toothed edge. Settlers were kept in containers in the laboratory for observation rather than being placed on the reef where they risked predation, bacterial attack, or trampling by small invertebrates. We assumed that they would not survive well under laboratory conditions. However, the settler attached to the plastic container survived for nearly two months. Even more remarkably, the settlers on the pre-cured ceramic surfaces survived for over seven months and showed a clear increase in tentacle length (Figure 5B) before eventually dying. No polyp division was observed in any of the settlers.

## Discussion

### Predictable spawning times across months and years

For the threatened Caribbean pillar coral *Dendrogyra cylindrus*, we report consistent spawning times across three consecutive years and across five total lunar cycles (Figure 2). As in other Caribbean corals, spawning was closely synchronized with the lunar cycle and daily sunset times (e.g., [17,18]). Only two prior observations of *D. cylindrus* spawning are published, both from Florida: a single male colony was seen spawning at 112 minutes after sunset, three nights after the early August full moon in 2006 [9] and multiple males and females were seen spawning 95 to 110 minutes after sunset, three and four nights after the early August full moon in 2012 [10]. Our observations are consistent with these reports, though it appears that spawning in Curaçao occurs slightly later relative to sunset. We did not conduct monitoring in other months, nor did we monitor outside of nights 2 to 5 after the full moon. This leaves the possibility that additional *D. cylindrus* spawning occurs outside of the window documented here.

### Asynchronous spawning times of males and females

By tracking individual colony spawning times, we found that males consistently and predictably spawned earlier than females (Figure 2), an observation reported by Neely and colleagues [10], but which was not yet confirmed with individual colony data and statistical support. On some nights of our study, all observable male spawning ceased before any female spawning was seen.

After males began spawning, we observed some females with open mouths (Figure 3D), which is a rare behavior in corals. We also observed females exposing their eggs to ambient seawater though radial slits around the mouth (Figure 2D-G). As *D. cylindrus* colonies do not release eggs and sperm in synchrony, it is possible that some or all eggs are fertilized in situ, prior to release. The release of recently-fertilized zygotes or embryos is one variant of so-called “spermcasting,” a term that encompasses any fertilization strategy in marine invertebrates in which free-spawned sperm are used for internal fertilization [19]. For example, in some dioecious coral reef gorgonian species, free-spawned sperm are used by females to fertilize oocytes internally; females either promptly release these newly-fertilized embryos into the seawater or brood them externally on their surfaces for a number of days [20-23].

In scleractinian corals, researchers previously described males spawning before females in the dioecious Caribbean species *Stephanocoenia intersepta* (Blushing Star Coral) and *Montastraea cavernosa* (Great Star Coral; [18,24-26]). We previously presented photographic evidence supporting the possibility of internal fertilization in *S. intersepta* [26]. Further, when Hagman and colleagues collected eggs from females of both *M. cavernosa* and *S. intersepta*, they found surprisingly high rates of fertilization without adding any sperm [27]. This led the authors to suggest that these two so-called “broadcast spawners” were fertilized internally. Based on our observations, *D. cylindrus* appears to have both morphological and behavioral traits that would enable internal fertilization.

Regardless of the precise location of fertilization, the asynchronous release of gametes by males and females has the potential to increase individual fitness. One of Thorson’s rules is that, for dioecious marine invertebrates, males generally spawn before females [28]. By delaying reproductive investment until fertilization is likely, individual females may improve their overall rates of fertilization. Individual males may also benefit from spawning early and therefore dominating the available gamete pool under conditions of sperm competition (e.g., [29]). The apparent benefits of asynchronous spawning are reflected in the wide diversity of dioecious marine animal taxa, and even four genera of green algae, in which male spawning is known to occur before female spawning (e.g., [28,30,31]).

In sum, *D. cylindrus* is one of many dioecious marine broadcasters that have adopted a fertilization strategy other than synchronous male and female spawning. For now, the precise timing of fertilization in *D. cylindrus* and the window of gamete viability in the water column remain to be determined. Because there are currently no population genetic data from *D. cylindrus*, we cannot yet predict whether individuals of this species generally fertilize only their very close neighbors or whether gametes have the potential to survive dispersal and achieve fertilization over relatively long distances.

### Successful propagation of a threatened coral species

In the lab, we achieved the successful propagation of *D. cylindrus* larvae to the primary polyp settler stage. In the field, we documented physical characteristics for identifying gravid males and females on spawning nights. We also recorded the first cases of September spawning anywhere in this species’ range, thereby demonstrating that populations of this species can distribute reproductive effort across two consecutive months (so-called “split spawning”). The known, region-wide reproductive season for *D. cylindrus* now extends across three lunar cycles, from early August in Florida to late August and late September in the southern Caribbean.

In our propagation efforts, we only achieved successful larval development after we injected sperm underneath a watertight egg collection tent underwater. However, we could not determine the definitive timing of fertilization for these embryos because, once on shore, we combined approximately 30 eggs collected in this manner with additional sperm and with approximately 30 additional eggs collected underwater without this step. This was done to maximize gamete density and diversity in hopes that *any* egg would be fertilized. Ultimately, 28 of these 60 eggs in total underwent cell division and developed into larvae. It therefore remains possible that we had nearly 100% fertilization from the tented colony underwater, and 0% fertilization from gametes mixed on shore. Alternately, it is possible that lesser amounts of fertilization occurred both underwater and onshore. For researchers attempting to rear *D. cylindrus* larvae, we recommend collecting sperm underwater in syringes and transferring this directly to tented female colonies that either exhibit pre-spawning characteristics (Figure 2D-F) or that have been observed spawning on previous occasions. It also remains possible that researchers will succeed with the traditional method of mixing gametes onshore.

### The paradox of the missing juveniles

We found that rearing *D. cylindrus* larvae in the laboratory was relatively easy due to fast development and a short time to settlement competence. Further, settlers were surprisingly robust in the laboratory setting relative to *Orbicella* and *Acropora* spp., two other genera of spawning Caribbean corals that are listed as threatened. This presents a new paradox for the early life history of *D. cylindrus*: if settled primary polyps survive so well under relatively stressful conditions, why are sexually-produced recruits absent in all large-scale Caribbean reef surveys published over the past three decades? Given the fact that *D. cylindrus* extends its tentacles fully during the day, even small recruits (1 cm diameter) should be easily distinguished from other species in the Meandrinidae family, including those whose juveniles are similar to one another in appearance such as *Eusmilia fastigiata* and *Meandrina meandrites*.

If *D. cylindrus* settlers are not likely to be misidentified by researchers, what explains their absence in surveys? Are colonies experiencing low or failed fertilization (i.e., suffering from Allee effects due to mate limitation, because populations densities are below a critical threshold)? Are embryos or larvae highly sensitive to eutrophication or microbial attack in the water column? Are competent larvae missing a critical cue for settlement? Do settlers face a pathogen, predator, or competitor that causes extensive post-settlement mortality? Locating this population bottleneck is an important next step for conservation. Encouragingly, the advances we report here should help to make *D. cylindrus* a viable subject for research on coral early life history, and perhaps restoration, provided that gametes can be collected in sufficient numbers and that good fertilization rates are achieved. This is the first dioecious, spawning coral species in the Caribbean for which larval propagation methods have been described.

### Population biology of a threatened coral

Caribbean coral species suffer together through habitat destruction, overfishing, eutrophication, sewage, pollution, disease, and global climate change, yet *D. cylindrus* garners heightened concern because its particular life history characteristics, limited habitat preferences, and disease susceptibility together pose an additional threat to its viability [2,3]. Its listing as a threatened species is not due to its historical rarity per se, but rather due to these species-specific factors that disproportionately threaten the continued persistence of individuals. The peculiar life history characteristics of *D. cylindrus* do partly explain its low historical abundance, but more worryingly, these traits then further magnify its conservation plight on modern reefs by limiting recruitment and population growth. As far back as 1986, Szmant described the risk of local extinction for *D. cylindrus* due to its small population size, the rarity of small colonies, a limited geographic range, and the occasional occurrence of a lone colony in a vast area [8]. In addition, reproductive success is limited by dioecy [8,32], which reduces the number of potential mates relative to hermaphroditic species. Fragmentation [33], slow growth [15,33,34], and a long lifespan can create populations with many genetically identical individuals, among whom mating is impossible because fragments originating from one colony are all the same sex. Over the long term, extremely low sexual recruitment rates [11-14] also limit the introduction of new genetic diversity into a population.

We identified additional traits in *D. cylindrus* with potential consequences for population viability. Rapid development and fast settlement competence may affect average dispersal distances [35], thereby affecting population connectivity and local extinction risk. Importantly, our observations of fast development were not due to unusually high temperatures. We conducted our experiments at approximately ambient August/September seawater temperature in Curaçao. Over the past eight years in Curaçao, we have found that other spawning species such as *Acropora palmata* and *Orbicella faveolata* do develop slightly faster at warmer temperatures, but neither of these species have ever developed nearly as quickly as *D. cylindrus* did at the same temperature.

We observed some potential for long-distance dispersal in larvae that remained swimming for over four days. However, given the species’ virtually undetectable recruitment rates, this perhaps does more to explain the occurrence of lone colonies in vast reef swaths (e.g., Puerto Rico; [8], Barbuda; [K. Marhaver, unpublished data]) than it provides evidence that populations can rebound from local extinction or near-extinction on timescales relevant to ecology and conservation.

The occurrence of split-spawning likely affords male colonies an additional lunar cycle to produce gametes. However, the gametogenesis cycle for *D. cylindrus* females is reported to be three months long [8], meaning total annual population fecundity may not be increased by splitting reproduction over two consecutive months. Rather, split spawning could potentially reduce population viability if this results in gamete concentrations below the density required for fertilization on a given spawning night [36-38].

With *D. cylindrus* now officially listed as a threatened species by the U.S. Government, a history of scientific neglect burdens the conservation planning process. We still have no data on its size at sexual maturity or the relative contribution of sexual versus asexual reproduction to population dynamics. It remains possible that many dense stands of *D. cylindrus* are in fact made up of very few, or even single genotypes (e.g., [39]). Our knowledge gaps reduce the accuracy of population viability assessment, which is difficult even in well-studied corals due to their clonality, coloniality, fragmentation, and partial death [39-43]. Given these life history characteristics, demographic surveys of *D. cylindrus* and the assumption of long-distance dispersal may easily contribute to overestimates of population viability. This warrants a precautionary approach to conservation.

## Conclusions

Over three years and five lunar cycles, we documented the predictability of spawning times in the threatened Caribbean Pillar Coral *Dendrogyra cylindrus*. We also showed that spawning occurs over two consecutive months, expanding the window of opportunity for research on this coral’s reproductive biology. We successfully reared *D. cylindrus* larvae to the primary polyp settler stage for the first time, allowing for possible re-seeding trials using sexually-produced juveniles. We documented rapid development and a short time to settlement competence, which will facilitate research, but which can also have important consequences for larval dispersal and population connectivity. We also showed that males spawn before females, raising the possibility that some or all fertilization could occur internally. However, it remains unknown how long *D. cylindrus* gametes remain viable, how far larvae can disperse, and therefore how isolated existing populations are from one another. With male and female individuals spawning at different times in an already-rare species, the potential also exists for Allee effects to limit overall fertilization success, increasing the importance of protecting dense populations where they still occur. Overall, our research raises a new paradox. *D. cylindrus* larvae and settlers were remarkably robust in the laboratory, yet recruits are virtually absent from modern day reefs. Identifying the timing and cause of the underlying population bottleneck is an important next step. In the meantime, a precautionary approach to management is warranted. Looking forward, we hope that our advances in natural history and propagation will enable a new era of research on this threatened, understudied, and unique coral.

## Methods

### Study species and location

We observed *Dendrogyra cylindrus* (Pillar Coral) colonies during night dives in Curaçao (southern Caribbean) at depths of 5 to 8 m using SCUBA. Observations were made at the Sea Aquarium reef (12°4’59” N, 68°53’43” W) in August 2012, August 2013, and August 2014, and at the Water Factory reef (12°6’34” N, 68°57’23” W) in September 2013 and September 2014. Colonies that were monitored had at least one pillar that was at least 0.5 m tall. Smaller colonies and colony fragments were not monitored. At Sea Aquarium, two large stands of ~20 colonies were monitored. At the Water Factory site, up to 10 standalone colonies were monitored in total. Between 5-50% of colonies under observation were seen spawning in a given night. Data on observation dates, lunar cycles, and sunset times are presented in Table 1. These data along with individual colony spawning times are also provided in Additional file 1. All monitoring nights are included in both Figure 1 and Table 1. No monitoring was conducted in months other than August and September.

**Table 1:** **Moon and sun data for spawning observation dates in Curaçao, Southern Caribbean, August 2012 to September 2014**

**Full moon date and time (AST)**	**Observation date**	**Nights after full moon**	**Sunset time (AST)**	**Dive site**	**Observation window (MAS)**
31 Aug 2012	2 Sept	2 NAFM*	1845 AST	Sea Aquarium	112-145
0959 AST					
20 Aug 2013	23 Aug	3 NAFM	1851 AST	Sea Aquarium	84-144
2145 AST					
20 Aug 2013	24 Aug	4 NAFM	1851 AST	Sea Aquarium	109-159
2145 AST					
20 Aug 2013	25 Aug	5 NAFM	1850 AST	Sea Aquarium	110-166
2145 AST					
19 Sept 2013	22 Sept	3 NAFM	1832 AST	Water Factory	94-158
0713 AST					
10 Aug 2014	13 Aug	3 NAFM	1857 AST	Sea Aquarium	98-157
1410 AST					
10 Aug 2014	14 Aug	4 NAFM	1856 AST	Sea Aquarium	100-163
1410 AST					
8 Sep 2014	11 Sept	3 NAFM	1839 AST	Water Factory	96-166
2138 AST					

All times are listed as the local time in Willemstad, Curaçao (Atlantic Standard Time; AST). Observation windows are presented as minutes after sunset (MAS) and represent the window of time during which divers were directly observing *D. cylindrus* colonies. *Note that in Curaçao, the full moon on 31 August 2012 occurred early in the day (0959 AST), thus some researchers would choose to count 2 Sept 2012 as the third night after the full moon rather than the second.

### Gamete collection and fertilization methods

All scleractinian corals are regulated by CITES and *D. cylindrus* is listed as a threatened species by the U.S. Government [1]. In the research we report here, all field observations, collections, and experiments were carried out under the permissions and collecting permits granted to CARMABI by the Government of Curaçao (Ministry of Health, Environment, and Nature). Only gametes were collected during this project; no adult coral tissue or skeletal materials were removed from the reef.

To collect eggs from spawning female coral colonies, we constructed conical tents of polyester fabric (waterproof fabric shower curtain liners). Each tent was weighted on the bottom with pieces of limestone rubble. The top of each tent was attached to an inverted plastic funnel using nylon hex nuts and bolts. An inverted 50 mL polypropylene conical centrifuge tube (Falcon, Corning Life Sciences, Corning, NY) was installed on the narrow opening of each funnel. A hole was drilled (1.5 cm diameter) into each tube cap and the caps were secured in place with plastic tie-wraps and plastic tarp repair tape. Between uses, tents were rinsed in freshwater and left to dry in the sun while plastic tubes were cleaned with 10% bleach. Bleach was denatured by rinsing tubes in a dilute solution of sodium thiosulfate pentahydrate followed by three freshwater rinses.

On spawning nights, we placed conical tents over individual coral pillars or over entire small colonies. Released coral eggs were positively buoyant and accumulated in the conical tubes. Tubes were then removed from the tents, closed with new caps, and carried to shore by divers. We collected sperm from spawning male colonies using 60 mL and 500 mL plastic syringes, aiming for areas in the water column near spawning colonies where sperm density was visibly high, such as in the valley between two spawning pillars. Syringes were cleaned between each dive with a solution of 10% bleach. Residual bleach was denatured using a rinse in a dilute solution of sodium thiosulfate pentahydrate followed by three freshwater rinses.

To identify successful propagation methods, we used two different approaches to gamete collection and fertilization. In August 2012 and September 2013, we collected gametes separately from male and female colonies and mixed them on shore. In August 2013, we collected sperm from a spawning male colony and promptly released it underneath a tent that was placed over a female colony that had spawned the previous year. Eggs were collected from this tent at the end of the spawning period on the same night. Additional sperm and eggs were collected separately during the same dive. On shore, we combined approximately 30 eggs collected from the tented female with additional sperm and with approximately 30 additional eggs collected separately on the reef (not subject to in situ sperm addition). Thus, the resulting gamete pool contained eggs that we exposed to sperm in situ as well as eggs that had been collected without this step. All incubations for fertilization and development were performed in new, clear polystyrene clamshell deli containers with lids (volume ~1 L). Gametes were mixed at 2140 local time, approximately 22 minutes after the end of observed spawning.

With the embryo cohort from August 2013, we attempted three different incubation procedures after mixing gametes. For Treatment 1, 15 embryos were moved from the fertilization bin after 20 minutes and transferred to additional, unused sperm solution that had been collected on the reef. Embryos were incubated for an additional 90 minutes, then transferred to GF/F-filtered seawater (Whatman GF/F filter, GE Healthcare Bio-Sciences Corp., Piscataway, NJ). For Treatment 2, 25 embryos were moved from the fertilization bin after 20 minutes and placed in freshly-prepared filter-sterilized seawater (SSW; Sterivex GP 0.22 μm syringe filter, Millipore, Billerica, MA). For Treatment 3, 20 embryos were left in the original fertilization container for a total of 100 minutes and then transferred to SSW. We performed all manipulations with wide-bore, sterile plastic transfer pipettes to reduce the risk of damage to embryos from shearing forces. Water temperature was held at ambient seawater temperature (~29°C). Water circulation was maintained in each container by attaching airline tubing from an air pump to a glass Pasteur pipette, which was threaded into the container and aimed at the surface of the seawater.

Fully-developed, swimming larvae were offered settlement surfaces early in the larval stage, on the day after spawning, because the time to settlement competence was unknown. As settlement substrate, we used kiln stilts, i.e., ceramic tripods that are typically used to elevate pottery off of kiln shelves during firing (34 mm radius, AMACO, Indianapolis, IN). We previously found that these ceramic surfaces foster successful settlement by larvae of other coral species (M. Vermeij, unpublished data). Prior to use, some of these ceramic tripods were pre-cured for two months in a flow-through aquarium system where they developed a mature biofilm along with a community of crustose coralline algae and small amounts of turf algae. Before using the pre-cured tripods for larval settlement, we brushed them gently with a clean toothbrush to remove loose sediments and detritus. Larvae in Treatments 1 and 2 were offered one cured and one un-cured tripod, while larvae in Treatment 3 were offered only an un-cured tripod. Beginning on Day 4, we performed water changes regularly every 7 to 14 days using GF/F-filtered seawater. We examined containers and pottery tripods thoroughly for settlers on Days 4, 8, 16, and 23 after fertilization. We did not find any swimming larvae remaining after Day 23, therefore we only re-examined known settlers after that point. Laboratory air temperature was held so that water temperature remained at approximately 29°C. After Day 4, water was not circulated in the containers. Embryos and settlers were observed and photographed using a trinocular Nikon SMZ800 stereozoom microscope with a Canon G9 or Canon EOS Rebel T3i camera.

### Availability of supporting data

The data supporting the results of this article are presented in the manuscript and in Additional file 1.

## Additional file

Additional file 1:
***Dendrogyra cylindrus*** **spawning observations.** Data are included for all *D. cylindrus* spawning observations in Curaçao, Southern Caribbean, 2012–2014. This file includes data on observation date, number of nights after the full moon, local sunset time, dive site name, observation window, colony sex, unique colony ID number, and spawning start and stop times.

## Abbreviations

AST: Atlantic Standard Time
MAS: Minutes after sunset
NAFM: Nights after the full moon
SSW: Sterile seawater (Sterivex GP 0.22 μm syringe filter)

## References

[1] National Oceanic and Atmospheric Administration (2014). 79 FR 53851 - Endangered and threatened wildlife and plants: final listing determinations on proposal to list 66 reef-building coral species and to reclassify elkhorn and staghorn corals. Fed Reg.

[2] Brainard RE, Birkeland C, Eakin CM, McElhany P, Miller MW, Patterson M, et al. Status review report of 82 candidate coral species petitioned under the U.S. Endangered Species Act: NOAA Technical Memorandum NOAA-TM-NMFS-PIFSC-27; 2011. ᅟ

[3] Aronson R, Bruckner A, Moore J, Precht B, Weil E. *Dendrogyra cylindrus.* In: IUCN Red List of Threatened Species Version 2013.2*.* [www.iucnredlist.org]

[4] Kerr AM (2005). Molecular and morphological supertree of stony corals (Anthozoa: Scleractinia) using matrix representation parsimony. Biol Rev Cambridge Philos Soc.

[5] Fukami H, Chen CA, Budd AF, Collins A, Wallace C, Chuang YY, et al. Mitochondrial and nuclear genes suggest that stony corals are monophyletic but most families of stony corals are not (Order Scleractinia, Class Anthozoa, Phylum Cnidaria). PLoS ONE. 2008;3:e3222. 10.1371/journal.pone.0003222 PMC2528942 18795098

[6] Budd AF, Fukami H, Smith ND, Knowlton N (2012). Taxonomic classification of the reef coral family Mussidae (Cnidaria: Anthozoa: Scleractinia). Zool J Linn Soc.

[7] Fadlallah YH (1983). Sexual reproduction, development and larval biology in scleractinian corals. Coral Reefs.

[8] Szmant AM (1986). Reproductive ecology of Caribbean reef corals. Coral Reefs.

[9] Donahue S, Acosta A, Akins L, Ault J, Bohnsack J, Boyer J, et al. The state of coral reef ecosystems of the Florida Keys. In: Waddell JE, Clarke AM, editors. The State of Coral Reef Ecosystems of the United States and Pacific Freely Associated States: NOAA Technical Memorandum NOS NCCOS 73; 2008. p. ᅟ 161–87.

[10] Neely KL, Lunz KS, Macaulay KA (2013). Simultaneous gonochoric spawning of *Dendrogyra cylindrus*. Coral Reefs.

[11] Bak RPM, Engel MS (1979). Distribution, abundance and survival of juvenile hermatypic corals (Scleractinia) and the importance of life history strategies in the parent coral community. Mar Biol.

[12] Vermeij MJA, Bakker J, van der Hal N, Bak RPM (2011). Juvenile coral abundance has decreased by more than 50% in only three decades on a small Caribbean island. Diversity.

[13] Rogers CS, Fitz HC, Gilnack M, Beets J, Hardin J (1984). Scleractinian coral recruitment patterns at Salt River Submarine Canyon, St. Croix, U.S. Virgin Islands. Coral Reefs.

[14] Miller SL, Chiappone M, Rutten LM (2010). Abundance, distribution and condition of benthic coral reef organisms in the Upper Florida Keys National Marine Sanctuary – 2010 quick look report and data summary.

[15] Acosta A, Acevedo A (2006). Population structure and colony condition of *Dendrogyra cylindrus* (Anthozoa: Scleractinia) in Providencia Island, Colombian Caribbean. Proc 10th Int Coral Reef Symp.

[16] Irizarry-Soto E, Weil E (2009). Spatial and temporal variability in juvenile coral densities, survivorship and recruitment in La Paraguera, southwestern Puerto Rico. Caribb J Sci.

[17] Levitan DR, Fukami H, Jara J, Kline D, McGovern TM, McGhee KE, et al. Mechanisms of reproductive isolation among sympatric broadcast-spawning corals of the *Montastraea annularis* species complex. Evolution. 2004;58:308–23. 15068348

[18] Vize PD, Embesi JA, Nickell M, Brown DP, Hagman DK (2005). Tight temporal consistency of coral mass spawning at the Flower Garden Banks, Gulf of Mexico, from 1997–2003. Gulf Mex Sci.

[19] Bishop JDD, Pemberton AJ (2006). The third way: spermcast mating in sessile marine invertebrates. Integr Comp Biol.

[20] Benayahu Y, Loya Y (1983). Surface brooding in the red sea soft coral *Parerythropodium fulvum fulvum* (Forskål, 1775). Biol Bull.

[21] Brazeau DA, Lasker H (1990). Sexual reproduction and external brooding by the Caribbean gorgonian *Briareum asbestinum*. Mar Biol.

[22] Lasker HR, Kim K (1996). Larval development and settlement behavior of the gorgonian coral *Plexaura kuna* (Lasker, Kim and Coffroth). J Exp Mar Biol Ecol.

[23] Lasker H (2006). High fertilization success in a surface-brooding Caribbean gorgonian. Biol Bull.

[24] Hagman DK, Gittings SR, Vize PD (1998). Fertilization in broadcast spawning corals of the Flower Garden Banks National Marine Sanctuary. Gulf Mex Sci.

[25] Vize PD (2006). Deepwater broadcast spawning by *Montastraea cavernosa*, *Montastraea franksi*, and *Diploria strigosa* at the Flower Garden Banks, Gulf of Mexico. Coral Reefs.

[26] Vermeij MJA, Barrot KL, Johnson AE, Marhaver KL (2010). Release of eggs from tentacles in a Caribbean coral. Coral Reefs.

[27] Hagman DK, Gittings SR, Deslarzes KJP (1998). Timing, species participation, and environmental factors influencing annual mass spawning at the Flower Garden Banks (Northwest Gulf of Mexico). Gulf Mex Sci.

[28] Thorson G (1950). Reproductive and Larval Ecology of Marine Bottom Invertebrates.

[29] Levitan DR, McGovern TM. The Allee effect in the sea. In: Norse EA, Crowder LB, editors. Marine Conservation Biology: The Science of Maintaining the Sea’s Biodiversity. Washington, D.C.: Island Press; 2006. p. 47-57.

[30] Clifton KE (1997). Mass spawning by green algae on coral reefs. Science.

[31] Levitan DR, Birkhead TR, Moller AP (1998). Sperm limitation, sperm competition and sexual selection in external fertilizers. Sperm Competition and Sexual Selection.

[32] Richmond RH, Hunter CL (1990). Reproduction and recruitment of corals: comparisons among the Caribbean, the Tropical Pacific, and the Red Sea. Mar Ecol Prog Ser.

[33] Hudson JH, Goodwin WB (1997). Restoration and growth rate of hurricane damaged pillar coral (*Dendrogyra cylindrus*) in the Key Largo National Marine Sanctuary, Florida.. Proc 8th Int Coral Reef Symp.

[34] Hughes TP, Connell JH (1987). Population dynamics based on size or age? A reef-coral analysis. Am Nat.

[35] Gilmour J, Smith L, Brinkman R (2009). Biannual spawning, rapid larval development and evidence of self-seeding for scleractinian corals at an isolated system of reefs. Mar Biol.

[36] Knowlton N (2001). The future of coral reefs. Proc Natl Acad Sci U S A.

[37] Levitan DR (2004). Density-dependent sexual selection in external fertilizers: variances in male and female fertilization success along the continuum from sperm limitation to sexual conflict in the sea urchin *Strongylocentrotus franciscanus*. Am Nat.

[38] Lee AM, Sæther B-E, Engen S (2011). Demographic stochasticity, allee effects, and extinction: the influence of mating system and sex ratio. Am Nat.

[39] Baums IB, Devlin-Durante M, Laing BAA, Feingold J, Smith T, Bruckner A, et al. Marginal coral populations: the densest known aggregation of *Pocillopora* in the Galápagos Archipelago is of asexual origin. Front Mar Sci. 2014;1:1–11.

[40] Hughes TP (1984). Population dynamics based on individual size rather than age: a general model with a reef coral example. Am Nat.

[41] Hughes TP, Jackson JBC (1985). Population dynamics and life histories of foliaceous corals. Ecol Monogr.

[42] Miller KJ, Ayre DJ (2008). Population structure is not a simple function of reproductive mode and larval type: insights from tropical corals. J Anim Ecol.

[43] Vardi T, Williams DE, Sandin SA (2012). Population dynamics of threatened elkhorn coral in the northern Florida Keys, USA. Endang Spec Res.

